# Cell-Penetrating Peptoids: A Dual Functional Strategy for Upcoming Neuroregeneration Application

**DOI:** 10.1021/acschemneuro.5c00693

**Published:** 2025-09-15

**Authors:** Pratikshya Paudel, Rupali Kaur, Prabir Kumar Gharai

**Affiliations:** † Department of Chemistry, 7618 Oklahoma State University, Stillwater, Oklahoma 74078, United States; ‡ Department of Animal and Food Sciences, 7618 Oklahoma State University, Stillwater, Oklahoma 74078, United States

**Keywords:** Cell-penetrating peptoids, Human mesenchymal stem cells, Neurogenesis

## Abstract

Effective therapies for neurological conditions are a significant challenge since limited capacity of neurons for regeneration affects neuronal recovery. Despite their ineffective in vivo direct development into neurons, human mesenchymal stem cells (hMSCs) exhibit therapeutic promise through immunological regulation, neuroprotection, and improvement of endogenous repair. A promising strategy is to convert hMSCs into functional neurons using peptoids that also possess cell-penetrating abilities. Therefore, developing cell-penetrating peptoids that combine effective cellular uptake with neural differentiation induction could significantly progress neuroscience research and neuroregenerative treatments.

## Graphical abstract

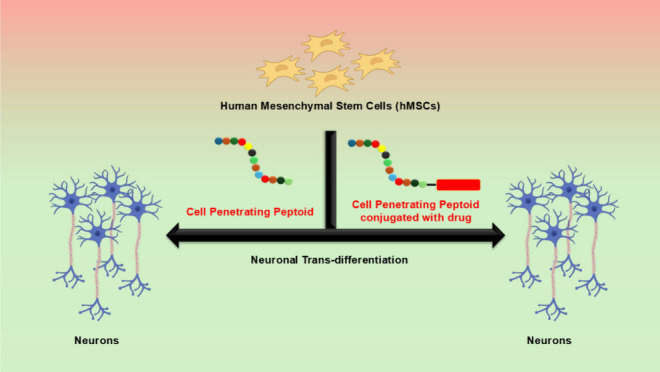

The process of neural development is highly regulated and includes the synchronization of programmed cell death, differentiation, and proliferation. The neural system undergoes neurogenesis throughout life, and it is crucial for memory, learning, and cognitive function. Stroke, Parkinson’s disease, Alzheimer’s disease, and Huntington’s disease are among the neurodegenerative diseases caused by the progressive and irreversible loss of neuronal structure and function. To treat such diseases, methods that encourage neuronal differentiation are crucial because neurons normally have a limited capacity for regeneration. In this field, therapeutic strategies include using cell-based therapies, which involve transplanting patients with differentiated neurons, or promoting endogenous neurogenesis, which generates new neurons in the adult nervous system.^1^

Most current therapeutics for neurological diseases emphasize symptom relief, underlining the critical need for treatments that can slow the development of the disease. Strategies based on stem cells, particularly those that use mesenchymal stem cells (MSCs), have a great chance of promoting neurological repair and delaying the progression of disease.^2,3^ MSCs are nonhematopoietic, multipotent stem cells that can self-renew and develop into multiple types of cells. They can be effectively grown in vitro for regenerative uses after being isolated from adult sources like bone marrow, adipose tissue, umbilical cord, and amniotic fluid. Their special capacity to migrate to injured tissues and have positive effects, such as regulating the immune response, promoting angiogenesis, offering neuroprotection, halting cell death, and lowering inflammation makes them promising therapeutic candidates for neurological disorders and injuries.^4^ MSCs have a lower risk of malignancy and are linked to fewer ethical issues than other forms of stem cells.

Human MSCs have been demonstrated to move toward damaged brain regions following transplantation, aiding in functional recovery. However, since very few transplanted cells survive in the brain and even fewer develop into neuronal cells, it is uncertain that this improvement is primarily due to the direct replacement of missing neurons through MSC development. However, the recovery effects are mostly attributed to paracrine pathways, which involve the release of bioactive substances that trigger the brain’s own recovery systems.^5^

Variability in treatment outcomes, heterogeneity in MSC sources and quality, and the requirement for optimized delivery systems are some of the obstacles that still exist despite positive advancements. It has been suggested that MSC survival, retention, and therapeutic potential can be improved by using biomaterials like peptides. Specifically in the context of allogeneic transplantation, further study is necessary to elucidate their in vivo mechanisms, establish standardized protocols, and promising safety. Developments in MSC biology, particularly in relation to their anti-inflammatory and immunomodulatory properties, are increasing the potential of these cells for customized regenerative treatments. To convert these experimental results into efficient therapies for neurodegenerative and neuroinflammatory diseases, executed clinical studies will be essential going forward.

Cell-penetrating peptides (CPPs) are short peptides that are effective in delivering drugs and genes because they can transport a variety of substances across the membranes of living cells. When it comes to delivering therapeutic compounds directly into cells and even through challenging biological barriers including the blood-brain barrier, skin, and ocular tissues, CPPs are crucial. This capability eliminates the need for painful injections and provides opportunities for noninvasive delivery systems including sprays, lotions, and ocular drops. When CPPs are combined with nanoparticles, they also become more stable, penetrate deeper into tissue, and handle better, which reinforces their function as a flexible platform for regenerative medicine and targeted therapy.

Nevertheless, because peptides are easily degraded by proteases and have poor cell permeability, their curative potential is frequently limited.^6^ In contrast, several peptoids have comparatively strong cell penetration and are resistant to enzymatic degradation. To facilitate the generation of peptoid libraries for future screening investigations, a high-throughput technique to assess the permeability of peptoids with different residues is highly beneficial.

Peptoid-based molecular transporters have become widely used over a decade because of their straightforward synthesis and exceptional resistance to enzymatic degradation. Diverse side chains can be incorporated due to their special structural flexibility. However, up to now, only lysine- or arginine-like motifs have been found in the cationic side chains of cell-penetrating peptoids. The goal of a recent study is to increase the variety of cationic side chains by first incorporating new functional groups into peptoid structures, such as polyamines, aza-crown ethers, and triphenylphosphonium ions.^6^ The hypothesis suggests that substituting peptide monomers with *N*-alkylglycine units in peptoids minimizes hydrogen-bonding potential, which is the fundamental reason responsible for their increased cell permeability.

Here we explained the advantages of a cell-penetrating peptoids (CPPds) over peptides for neuronal trans-differentiation from human mesenchymal stem cells (Figure 1). CPPds offer a novel platform for inducing the trans-differentiation of hMSCs into neurons. Peptoids are more stable than typical cell-penetrating peptides (CPPs) due to their N-substituted glycine backbone, which prevents proteolytic degradation. This characteristic ensures stability inside cells, allowing for uninterrupted supply of neurogenic substances. Furthermore, peptoids are extremely adaptable as their side chains can be changed to improve tissue selectivity, intracellular targeting, and endosomal escape. By conjugating a drug that can help neuronal trans-differentiation or transcription factors such as NeuroD1, ASCL1, or Ngn2 to CPPds may be possible to directly activate neuronal gene networks in hMSCs. Moreover, the structural diversity of peptoids enables the design of multifunctional carriers that combine targeting, delivery, and release properties in a single molecule. These features could improve both the efficiency and safety of reprogramming approaches for neuroregenerative therapies. CPPds may also overcome the limitations of CPPs in vivo, such as rapid degradation and off-target effects. Altogether, CPPd-based strategies hold significant promise as a stable, versatile, and clinically translatable approach for promoting hMSC-to-neuron trans-differentiation. Therefore, the dual function strategies of CPPds not only deliver the drugs into cells but also helps directly neuronal trans-differentiation from hMSCs will be important in future neuroregenerative research.

**Figure 1:**
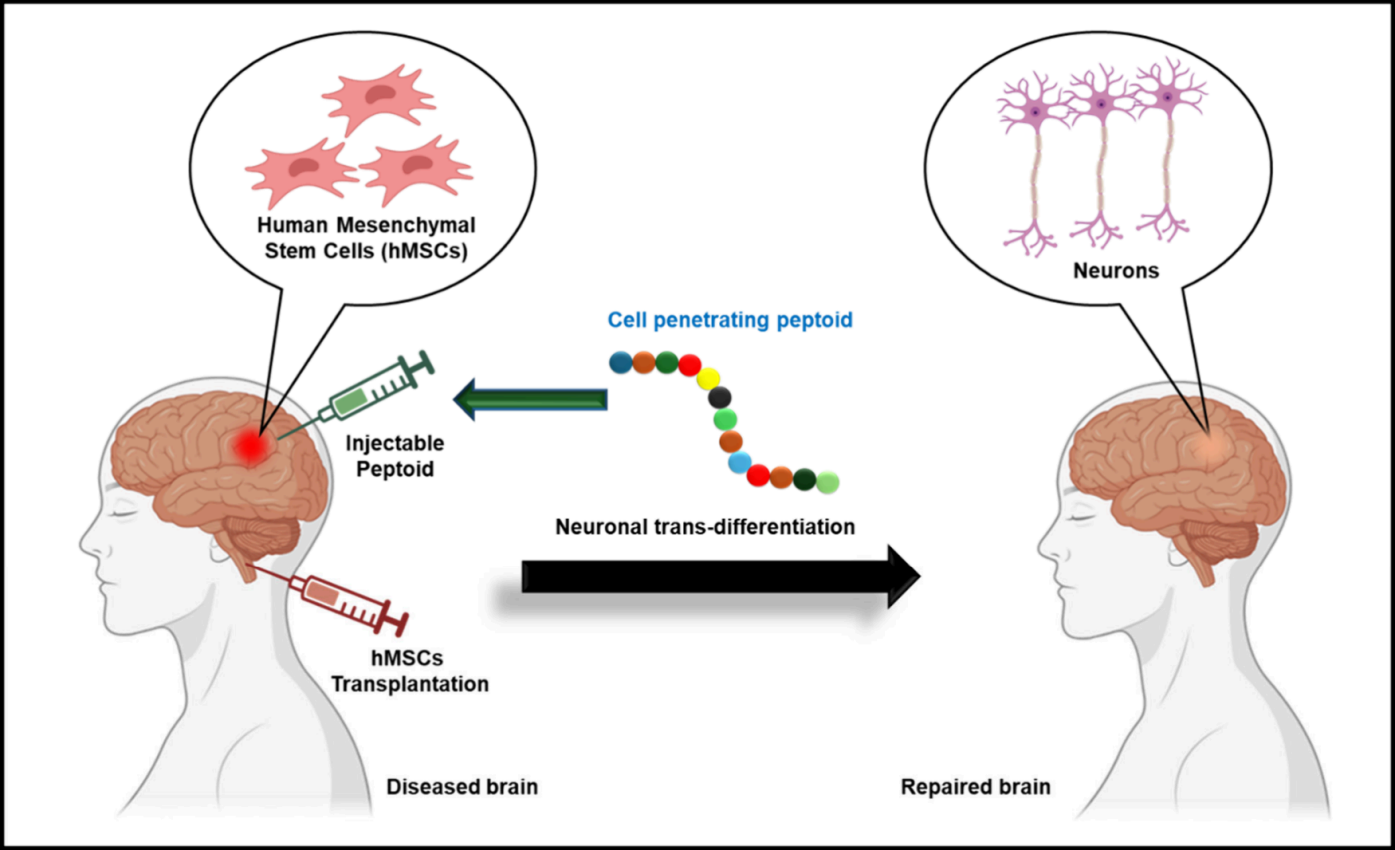
Schematic representation of cell penetrating peptoid-based approach that promotes neuronal trans-differentiation from hMSC.

In conclusion, many peptides that drive stem cell differentiation toward neurons lack efficient cell-penetrating ability, whereas peptoids, which are resistant to proteolysis, have not been reported to simultaneously promote neuronal differentiation and possess cell-penetrating properties. This gap underscores a critical limitation in current neuroregenerative approaches. Developing cell-penetrating peptoids capable of both inducing neuronal trans-differentiation and efficiently entering cells would constitute a major advancement in the field. Such multifunctional biomolecules could revolutionize therapeutic interventions and provide new tools for studying the mechanisms underlying neurodegenerative diseases.
